# Does Parenteral Iron Increase the Risk of Infection in Patients with Gastrointestinal Cancer? A Systematic Review and Meta-Analysis

**DOI:** 10.3390/cancers18121859

**Published:** 2026-06-06

**Authors:** Saikat Mandal, Madeleine Taylor, Aswin K Mohan, Shirin R. Hasan, Manideepa Maji, Melek-Mary Aydin, Suhasini Sil, Arkadeep Dhali

**Affiliations:** 1 Nottingham Digestive Diseases Centre, Translational Medical Sciences, School of Medicine, University of Nottingham, Nottingham NG7 2UH, UK; saikat.mandal@nottingham.ac.uk; 2 NIHR Nottingham Biomedical Research Centre, Nottingham University Hospitals NHS Trust and the University of Nottingham, Nottingham NG7 2UH, UK; 3 School of Medicine, University of Nottingham, Nottingham NG7 2UH, UK; mzymt20@nottingham.ac.uk (M.T.); mzyma38@nottingham.ac.uk (M.-M.A.); 4 All India Institute of Medical Sciences, Bibinagar 508126, India; aswinakmakm@gmail.com; 5 Queen’s Centre for Oncology and Haematology, Hull University Teaching Hospitals NHS Trust, Hull HU16 5JQ, UK; shirin.h2017@gmail.com; 6 Hull York Medical School, University of Hull, Hull HU6 7RX, UK; manideepa.maji@hyms.ac.uk; 7 Haematology, Hull University Teaching Hospitals NHS Trust, Hull HU16 5JQ, UK; 8 All India Institute of Medical Sciences, New Delhi 110029, India; suhasinisil28nov@gmail.com; 9 Academic Unit of Gastroenterology, Sheffield Teaching Hospitals NHS Foundation Trust, Sheffield S5 7AU, UK; 10 School of Medicine, Dentistry and Biomedical Sciences, Queen’s University Belfast, Belfast BT9 7BL, UK

**Keywords:** parenteral iron, intravenous iron, gastrointestinal cancer, colorectal cancer, infection, systematic review, meta-analysis

## Abstract

**Background**: Parenteral iron is increasingly used to treat iron deficiency anaemia in patients with gastrointestinal cancer, particularly in the perioperative setting. However, concerns persist that intravenous iron could increase the risk of infection. We conducted a systematic review and meta-analysis to evaluate infectious complications and other relevant clinical outcomes after parenteral iron in gastrointestinal cancer. **Methods**: Embase, MEDLINE, CENTRAL and Scopus were searched from database inception to 20 February 2026, with additional records identified through citation searching. Comparative studies were eligible if they included adults with gastrointestinal cancer and compared parenteral iron with oral iron, placebo, no iron or standard care. Randomised and non-randomised comparative studies were included. The primary outcome was infection or infective complications. Secondary outcomes included length of hospital stay, hospital readmission and short-term mortality. Risk ratios (RRs) and mean differences (MDs) were pooled using random-effects models. **Results**: The search identified 2771 records. After removing 458 duplicates, 2313 records were screened, and 210 full texts were assessed. Fourteen comparative studies were included: four randomised controlled trials and ten non-randomised studies. Nine studies contributed to the primary infection analysis, including 899 patients receiving parenteral iron and 768 controls. Infection occurred in 126 patients in the parenteral iron group and 104 controls. Parenteral iron was not associated with an increased risk of infection (RR 1.07, 95% CI 0.71–1.62; *p* = 0.754; I^2^ = 57%). Restriction to randomised trials also showed no significant increase in infection risk (RR 1.17, 95% CI 0.56–2.43). Thirteen studies reported length of stay, with no meaningful difference between groups (MD: −0.05 days, 95% CI: −0.66 to 0.57). Four studies reported hospital readmission, with no significant difference between parenteral iron and control (RR 0.76, 95% CI 0.35–1.64; *p* = 0.480; I^2^ = 0%). Mortality analyses were event-limited, with no significant difference in 30-day mortality (RR 0.49, 95% CI 0.09–2.61) or 90-day mortality (RR 0.69, 95% CI 0.35–1.36). **Conclusions**: In patients with gastrointestinal cancer, parenteral iron was not associated with increased infection risk, prolonged hospital stay, higher readmission or increased short-term mortality. These findings provide reassuring evidence regarding the perioperative safety of parenteral iron, although confidence in the estimate is limited by observational evidence and heterogeneous definitions of infection.

## Simple Summary

Anaemia and iron deficiency are common in people with gastrointestinal cancer. Anaemia can affect energy levels, recovery, ability to tolerate cancer treatment, and overall outcomes. Iron tablets are often difficult to tolerate and may not work quickly enough, so iron given directly into a vein is increasingly used. However, some clinicians worry that this treatment could increase the risk of infection because iron can also support the growth of certain bacteria. We reviewed and combined available studies comparing intravenous iron with iron tablets, no iron treatment, or usual care in patients with gastrointestinal cancer. Overall, intravenous iron was not associated with a higher risk of infection, longer hospital stay, hospital readmission, short-term death, or surgical wound infection. These findings are reassuring for clinicians and patients considering intravenous iron, but the evidence is still limited by differences between studies and incomplete infection reporting. Future studies should report infection outcomes more consistently, clearly and transparently.

## 1. Introduction

Anaemia is common in patients with gastrointestinal cancer and is particularly relevant in colorectal and gastric cancer surgery. Its aetiology is often multifactorial, reflecting chronic tumour-related blood loss, reduced dietary intake, malabsorption, systemic inflammation and impaired iron mobilisation through hepcidin-mediated pathways [1,2,3]. In surgical patients, anaemia may be further compounded by perioperative blood loss and postoperative inflammation, leading to delayed recovery, reduced functional status and greater need for red blood cell transfusion [4,5]. Iron deficiency is one of the most important reversible contributors to anaemia in gastrointestinal cancer. Optimising haemoglobin and iron stores is therefore a central component of patient blood management, particularly before major cancer surgery [6,7].

Oral iron is often used because it is inexpensive and readily available. But its effectiveness may be limited in gastrointestinal cancer due to poor tolerance, impaired absorption, inflammation-driven hepcidin upregulation, and the short interval between cancer diagnosis and surgery [8,9]. Parenteral iron offers a practical alternative because it bypasses the gastrointestinal tract and can replenish iron stores more rapidly [7]. Several studies have reported improved haemoglobin recovery after intravenous iron in selected gastrointestinal cancer populations, although effects on red blood cell transfusion have been inconsistent across trials and observational cohorts [10,11,12]. This inconsistency reflects important clinical and methodological differences, including cancer site, timing of iron administration, iron formulation, comparator strategy, baseline anaemia severity, transfusion thresholds and perioperative care pathways.

Despite these potential benefits, concerns remain regarding the safety of parenteral iron in patients with cancer. Iron is an essential nutrient for many pathogens, and host iron trafficking is closely linked to innate immune responses. These mechanisms underpin theoretical concerns that parenteral iron could increase susceptibility to infection [13,14]. A previous meta-analysis across broader clinical populations also reported an increased infection signal after intravenous iron, although this was not specific to gastrointestinal cancer [15]. This issue is clinically relevant in patients with gastrointestinal cancer, where infectious complications, including surgical site infection, pneumonia, urinary tract infection, intra-abdominal abscess and sepsis, contribute substantially to morbidity and prolonged hospitalisation. Postoperative complications may delay adjuvant oncological treatment [16,17,18,19]. Furthermore, untreated anaemia and allogeneic blood transfusion may themselves be associated with adverse postoperative outcomes, including infection [4,5].

Existing evidence regarding the association between parenteral iron and infection in gastrointestinal cancer surgery remains heterogeneous. Randomised trials have often been powered to evaluate haemoglobin response or transfusion outcomes rather than infectious outcomes, while observational studies vary in study design, timing of iron administration, comparator groups, cancer populations and outcome definitions. Furthermore, reported outcomes vary across studies, with some evaluating overall infectious complications and others reporting only individual specific events such as wound infection, pneumonia, or urinary tract infection. Consequently, the overall effect of parenteral iron on the risk of clinically relevant infection in this population remains uncertain.

We therefore conducted a systematic review and meta-analysis of randomised and non-randomised comparative studies evaluating parenteral iron in patients with gastrointestinal cancer. The primary objective was to determine whether parenteral iron is associated with an increased risk of infection or infectious complications compared with oral iron, placebo, no iron or standard care. Secondary objectives were to evaluate its association with length of hospital stay, hospital readmission, short-term mortality and surgical site infection.

## 2. Methods

### 2.1. Design and Reporting

This systematic review and meta-analysis were conducted and reported in accordance with the Preferred Reporting Items for Systematic Reviews and Meta-Analyses (PRISMA) 2020 statement and methodological principles from the Cochrane Handbook for Systematic Reviews of Interventions [20,21] (Table S1). The review was prospectively registered with PROSPERO (CRD420261289224).

Randomised and non-randomised comparative studies were included because the available evidence base in gastrointestinal cancer includes both trial and real-world perioperative cohorts.

### 2.2. Search Strategy and Information Sources

Embase, MEDLINE, CENTRAL and Scopus were searched from database inception to 20 February 2026. Additional records were identified through citation searching. The search combined controlled vocabulary and free-text terms for gastrointestinal malignancy, colorectal cancer, gastric cancer, oesophageal or oesophagogastric cancer, parenteral or intravenous iron, infection, postoperative complications and perioperative outcomes. Search results were imported into Covidence for de-duplication and screening. Full database-specific search strategies are provided in Appendix A.

Titles and abstracts were screened independently by two reviewers, with disagreements resolved by discussion or a third reviewer. Full-text eligibility assessment, data extraction and risk-of-bias assessment were performed independently by two reviewers using prespecified forms. Any discrepancies were resolved by consensus.

### 2.3. Eligibility Criteria

Studies were eligible if they included adults with gastrointestinal cancer and compared the intervention, parenteral iron, with oral iron, placebo, no iron, observation or standard care. Gastrointestinal cancers included colorectal, colon, rectal, gastric, oesophageal and gastro-oesophageal cancers. Mixed-cancer studies were included only if outcome data were separately extractable for the gastrointestinal cancer subgroup.

Eligible designs were randomised controlled trials, quasi-randomised trials, prospective and retrospective comparative cohort studies, before-and-after studies, and propensity score-matched observational studies. Case reports, single-arm case series, narrative reviews, editorials, protocols without outcome data and conference abstracts without extractable arm-level data were excluded from quantitative synthesis. Studies were not excluded solely for not reporting the primary infection outcome. If an otherwise eligible study reported extractable secondary outcomes, it was retained for those analyses and infection was recorded as not reported.

Reports describing the same study population were collated under the parent study to avoid double-counting. Studies in which all groups received parenteral iron were excluded from the comparative meta-analysis because they did not provide a valid parenteral iron versus non-parenteral iron comparison.

### 2.4. Outcomes

The primary outcome was infection or infective complications, extracted as the number of patients with infection in each treatment arm. Infection outcomes included overall infection, surgical site or wound infection, pneumonia or respiratory tract infection, urinary tract infection, intra-abdominal abscess, sepsis, unidentified infection, catheter-related infection and other infective complications as defined by the study authors. Where a total infection count was not reported but individual infective complications were available, these were extracted separately, and a derived total infection count was calculated by summing infective subtypes. Such estimates were labelled as imputed and considered potentially at risk of double-counting if the same participant could experience more than one infective event. Fever alone was not counted as an infection unless explicitly defined as an infective complication. Anastomotic leak, intra-abdominal collection, or wound dehiscence were not counted as infection unless reported as infection or described as abscess, sepsis, or microbiologically/radiologically confirmed infection. Secondary outcomes included length of hospital stay, hospital readmission, 30-day and 90-day all-cause mortality, and surgical site/wound infection.

### 2.5. Data Extraction and Risk of Bias

Data were extracted into a prespecified spreadsheet by study arm. Extracted variables included study design, country, cancer site, sample size, intervention formulation and dosing, comparator, timing of iron administration, infection outcomes, length of stay, readmission, mortality and adverse events. Dichotomous outcomes were extracted as event counts and denominators. Continuous outcomes were extracted as mean, standard deviation and sample size; where studies reported medians and interquartile ranges, these were retained and converted for meta-analysis using established methods [22].

Risk of bias was assessed using RoB 2 for randomised trials and ROBINS-I for non-randomised comparative studies [23,24]. For RoB 2, domains included randomisation, deviations from intended interventions, missing outcome data, measurement of the outcome and selection of the reported result. For ROBINS-I, domains included confounding, selection of participants, intervention classification, deviations from intended interventions, missing data, outcome measurement, and selection of the reported result. Risk-of-bias judgements were not used as exclusion criteria, but were considered when interpreting the confidence and robustness of the findings.

### 2.6. Data Synthesis and Statistical Analysis

Meta-analysis was undertaken when at least three studies reported an outcome with extractable arm-level data. For dichotomous outcomes, risk ratios (RRs) with 95% confidence intervals (CIs) were calculated and pooled on the natural-log scale. A continuity correction of 0.5 was applied when a study contained one or more zero cells. For continuous outcomes, mean differences (MDs) with 95% CIs were pooled directly. Median and interquartile range data were converted to approximate mean and standard deviation values using the method of Wan and colleagues [22].

Random-effects models using inverse-variance weighting and the DerSimonian–Laird estimator were used as the primary analyses because clinical heterogeneity was expected across cancer sites, iron formulations, timing of iron administration and comparator strategies [25]. Between-study heterogeneity was assessed using τ^2^, Cochran Q and the I^2^ statistic [26]. Analyses were implemented in Python 3 using NumPy and SciPy, with forest plots generated in R using the *meta* package and funnel plots produced in Python using matplotlib.

### 2.7. Sensitivity and Subgroup Analysis

Prespecified sensitivity analyses for the primary outcome excluded studies with imputed infection counts and restricted the analysis to randomised controlled trials. Additional subgroup and sensitivity analyses included non-randomised studies only, a leave-one-out analysis for infection, exclusion of non-randomised studies at serious risk of bias, and a length-of-stay analysis restricted to studies that reported mean and standard deviation directly. A post hoc sensitivity analysis using REML with Hartung–Knapp adjustment was also undertaken for the primary infection outcome. Subgroup analyses were undertaken, where sufficient extractable arm-level data were available, according to cancer site, iron formulation and timing of parenteral iron administration. For subgroup analyses, subgroup-specific estimates were pooled descriptively when at least two studies contributed data; formal subgroup-difference tests were performed only when both subgroups had at least two studies.

### 2.8. Publication Bias

Small-study effects and potential publication bias were explored using funnel plots and Egger’s test for outcomes with sufficient contributing studies. For the primary infection outcome, this assessment was considered exploratory because only nine studies contributed to the analysis.

## 3. Results

### 3.1. Study Selection

**Figure 1:**
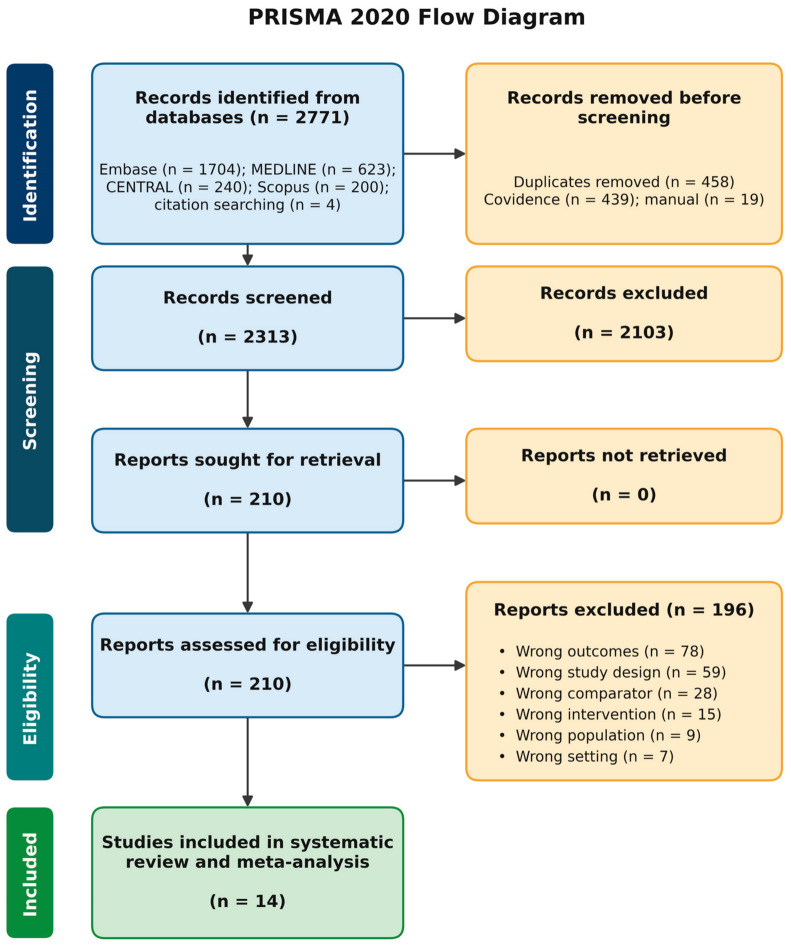
PRISMA flow diagram for study selection.

The search identified 2771 records: Embase (*n* = 1704), MEDLINE (*n* = 623), CENTRAL (*n* = 240), Scopus (*n* = 200) and citation searching (*n* = 4). After removing 458 duplicates, 2313 records were screened, and 210 full texts were assessed. Fourteen unique comparative studies were included in the systematic review and meta-analysis (Figure 1).

### 3.2. Study Characteristics

The 14 included studies comprised four randomised controlled trials and ten non-randomised comparative studies. Studies were published between 2014 and 2024 and conducted in South Korea, Spain, the UK, Hong Kong, Finland, Denmark, the Netherlands and Greece. Most studies enrolled patients undergoing surgery for colorectal or colon cancer, while three included patients with gastric cancer. Parenteral iron was administered preoperatively, postoperatively or during perioperative care, and control groups received oral iron, placebo, observation, no iron, or standard care. Key characteristics are summarised in Table 1.

### 3.3. Risk of Bias

**Figure 2:**
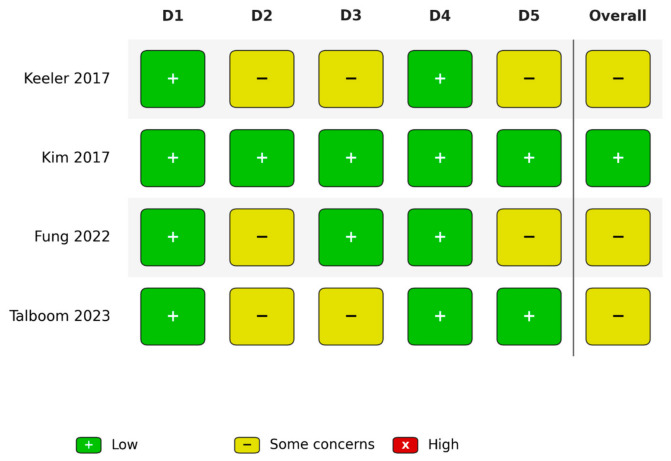
**Risk-of-bias assessment for randomised controlled trials using RoB 2.** Traffic-light plot showing domain-level and overall risk-of-bias judgements for the included randomised controlled trials. D1 = bias arising from the randomisation process; D2 = bias due to deviations from intended interventions; D3 = bias due to missing outcome data; D4 = bias in measurement of the outcome; D5 = bias in selection of the reported result. Green indicates low risk of bias, yellow indicates some concerns, and red indicates high risk of bias.

**Figure 3:**
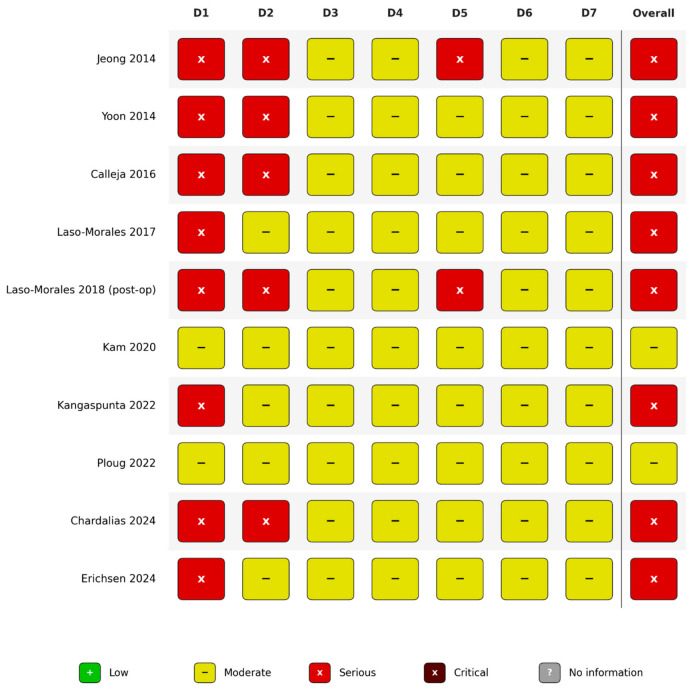
**Risk-of-bias assessment for non-randomised comparative studies using ROBINS-I.** Traffic-light plot showing domain-level and overall risk-of-bias judgements for the included non-randomised comparative studies. D1 = bias due to confounding; D2 = bias in selection of participants into the study; D3 = bias in classification of interventions; D4 = bias due to deviations from intended interventions; D5 = bias due to missing data; D6 = bias in measurement of outcomes; D7 = bias in selection of the reported result. Green indicates low risk of bias, yellow indicates moderate risk of bias, red indicates serious risk of bias, dark red indicates critical risk of bias, and grey indicates no information.

Among the four randomised trials, one was judged to be at low overall risk of bias. In contrast, three were judged to have some concerns, most commonly related to open-label design, possible deviations from intended interventions, or incomplete reporting of prespecified outcomes (Figure 2). Among the ten non-randomised comparative studies, two were judged to be at moderate overall risk of bias and eight at serious risk of bias (Figure 3). Serious risk-of-bias judgments were driven primarily by confounding, non-random treatment allocation, and selection of participants into intervention pathways. No non-randomised study was judged to be at critical risk of bias. Because most non-randomised studies were judged to be at serious risk of bias, pooled observational estimates were interpreted as supportive rather than definitive.

### 3.4. Primary Outcome: Infection or Infective Complications

**Figure 4:**
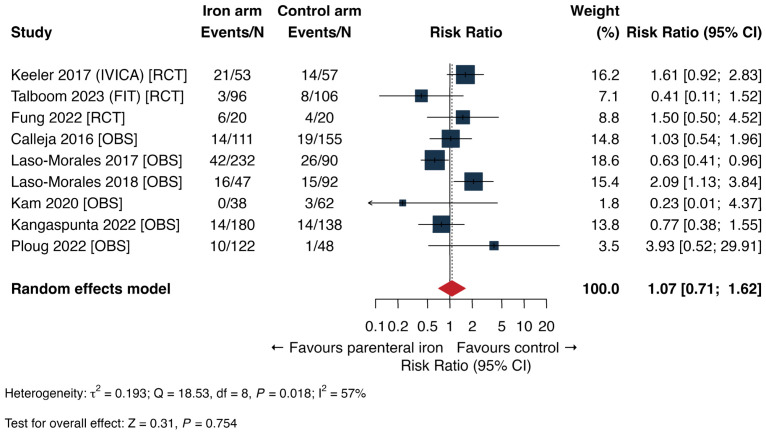
Primary outcome: infection or infective complications after parenteral iron in patients with gastrointestinal cancer.

Nine of the 14 included studies reported or allowed imputation of the number of patients experiencing at least one infectious complication following parenteral iron versus control. Across these studies, infection occurred in 126 of 899 patients receiving parenteral iron and 104 of 768 controls. Parenteral iron was not associated with an increased risk of infection (random-effects RR 1.07, 95% CI 0.71 to 1.62; *p* = 0.754). Statistical heterogeneity was moderate (τ^2^ = 0.193; Q = 18.53, df = 8, *p* = 0.018; I^2^ = 57%). The primary analysis is shown in Figure 4.

### 3.5. Sensitivity and Subgroup Analyses for Infection

**Figure 5:**
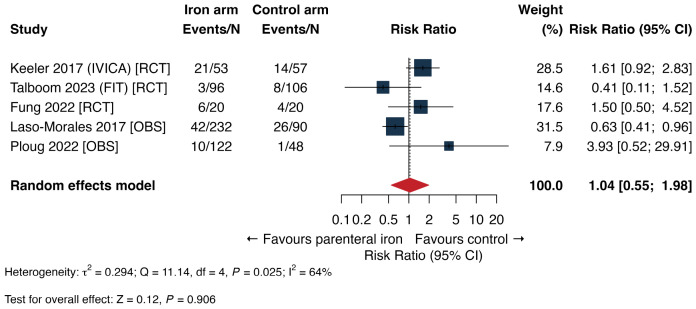
Sensitivity analysis: infection outcome excluding studies with imputed infection events.

**Figure 6:**
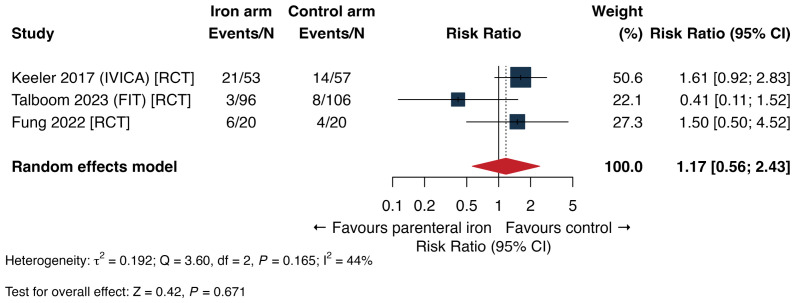
Sensitivity analysis: infection outcome restricted to randomised controlled trials.

Four studies required imputation of infection events from reported infective subtypes or percentages. Exclusion of these studies did not materially alter the pooled estimate (RR 1.04, 95% CI 0.55 to 1.98; *p* = 0.906; I^2^ = 64%; Figure 5). Restriction of the analysis to randomised trials likewise showed no significant increase in infection risk (RR 1.17, 95% CI 0.56 to 2.43; *p* = 0.671; I^2^ = 44%; Figure 6). Findings from the non-randomised-only subgroup analysis, risk-of-bias sensitivity analysis and the post hoc REML with Hartung–Knapp sensitivity analysis were also consistent with the primary analysis (Table 2).

Leave-one-out analysis showed that no single study materially changed the primary conclusion. The pooled estimate remained non-significant after removing each study in turn (Table 3).

### 3.6. Secondary Outcomes

#### Length of Hospital Stay

**Figure 7:**
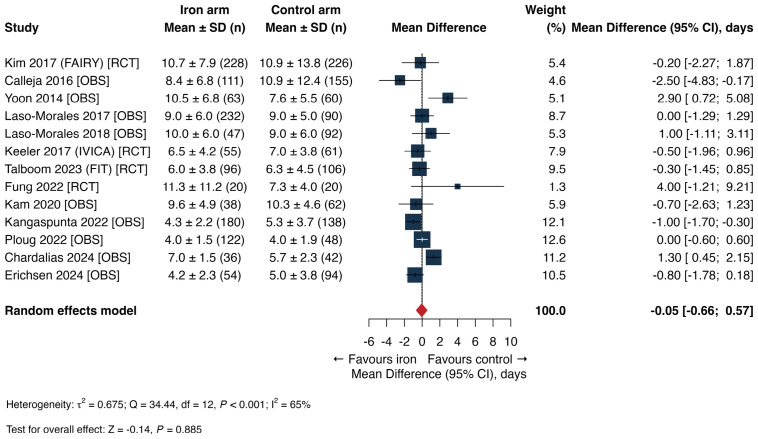
Secondary outcome: length of hospital stay after parenteral iron.

Thirteen studies contributed to the length-of-stay analysis. Parenteral iron was not associated with a significant difference in hospital length of stay (MD −0.05 days, 95% CI −0.66 to 0.57; *p* = 0.885), although substantial heterogeneity was observed (τ^2^ = 0.675; Q = 34.44, df = 12, *p* < 0.001; I^2^ = 65%; Figure 7). A sensitivity analysis restricted to studies that directly reported the mean and standard deviation was consistent with the primary analysis (Table 4).

### 3.7. Hospital Readmission

**Figure 8:**
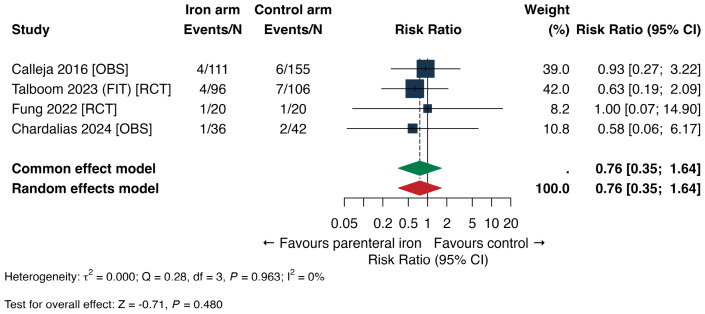
Secondary outcome: hospital readmission after parenteral iron.

Four studies reported hospital readmission, comprising 263 patients receiving parenteral iron and 323 controls. Readmission occurred in 10 patients in the parenteral iron group and 16 in the control group, with no significant between-group difference (RR 0.76, 95% CI 0.35 to 1.64; *p* = 0.480; I^2^ = 0%; Figure 8).

### 3.8. Short-Term Mortality

**Figure 9:**
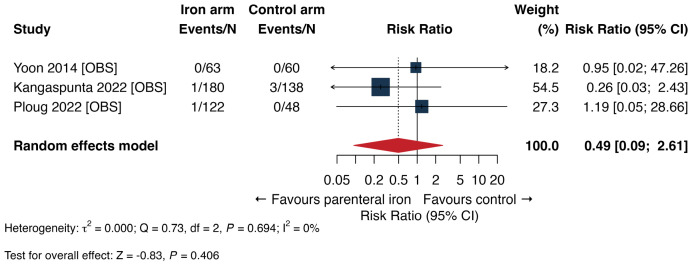
Secondary outcome: 30-day all-cause mortality after parenteral iron.

**Figure 10:**
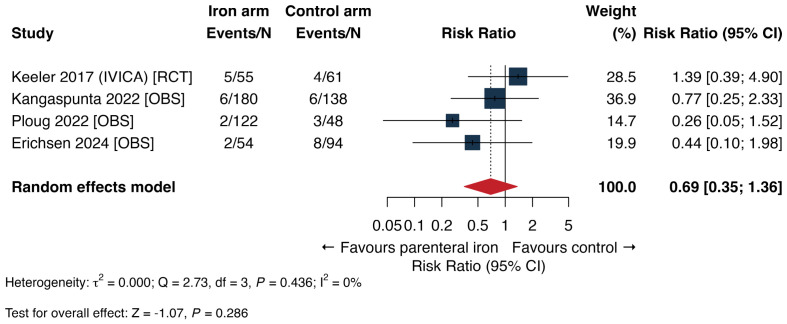
Secondary outcome: 90-day all-cause mortality after parenteral iron.

Mortality analyses were limited by low event rates and were therefore stratified by follow-up interval. Three studies reported 30-day all-cause mortality. Across these studies, two of 365 patients receiving parenteral iron and three of 246 controls died within 30 days (RR 0.49, 95% CI 0.09 to 2.61; *p* = 0.406; I^2^ = 0%; Figure 9). Four studies reported 90-day all-cause mortality. Across these studies, 15 of 411 patients in the parenteral iron arm and 21 of 341 controls died within 90 days (RR 0.69, 95% CI 0.35 to 1.36; *p* = 0.286; I^2^ = 0%; Figure 10).

### 3.9. Surgical Site/Wound Infection

**Figure 11:**
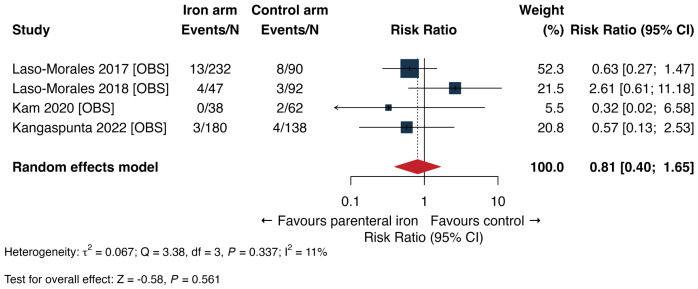
Surgical site/wound infection after parenteral iron in patients with gastrointestinal cancer.

Four observational studies reported extractable arm-level data for surgical site or wound infection as a specific infective complication (Figure 11). Across these studies, surgical site/wound infection occurred in 20 of 497 patients receiving parenteral iron and 17 of 382 controls. Parenteral iron was not associated with a statistically significant increase in surgical site/wound infection risk (random-effects RR 0.81, 95% CI 0.40 to 1.65; *p* = 0.561), with low between-study heterogeneity (τ^2^ = 0.067; Q = 3.38, df = 3, *p* = 0.337; I^2^ = 11%). This analysis was considered exploratory because only a small number of studies reported this infection subtype, and all contributing studies were observational. No included study reported extractable arm-level data on intraoperative surgical-site contamination or operative-field contamination; therefore, this could not be meta-analysed separately.

### 3.10. Synthesis of Findings

Overall, the available evidence did not demonstrate an increase in infection, hospital readmission, prolonged length of stay or short-term mortality following parenteral iron in patients with gastrointestinal cancer (Table 5). The infection analysis remained consistent across multiple sensitivity analyses, including exclusion of imputed infection events, restriction to randomised trials, restriction to non-randomised studies, leave-one-out testing, and risk-of-bias sensitivity analysis. However, most included studies were observational, definitions of infection varied across studies, and mortality analyses were limited by low event rates. These limitations reduce confidence in the findings despite the consistency of effect estimates across sensitivity analyses.

The cancer site was assessed as a subgroup variable (Table 6) where sufficient data were available. Only the length-of-stay outcome included both colorectal/colon and gastric cancer studies and could therefore be compared by cancer site. For infection, hospital readmission and 90-day mortality, only colorectal/colon cancer studies contributed extractable arm-level data. For 30-day mortality, two colorectal/colon studies and one gastric study reported data; however, the gastric subgroup comprised a single zero-event study and was not suitable for pooled subgroup estimation. The gastric length-of-stay subgroup included only two studies and should therefore be interpreted cautiously.

Iron formulations used in the included studies (Table 7) were grouped into two categories: ferric carboxymaltose (administered as a high-dose single or limited-dose-number infusion) and other formulations (iron sucrose, ferric derisomaltose/iron isomaltoside, or mixed formulations within the same study cohort). Studies in which different formulations were used within the same arm were classified as non-ferric carboxymaltose to provide a conservative comparator group. Test for subgroup difference between the two formulation categories was assessed by the Cochran Q-between statistic on one degree of freedom.

The timing of parenteral iron administration (Table 8) was assessed as an additional exploratory subgroup variable. Studies were classified as preoperative (iron administered before surgery; n = 9 of 14 included studies) or postoperative (iron administered after surgery; n = 5 of 14). Subgroup-pooled estimates and tests for subgroup differences (Cochran Q-between, df = 1) are reported below for each outcome in which both subgroups had at least two contributing studies.

### 3.11. Reporting of Publication Bias

**Figure 12:**
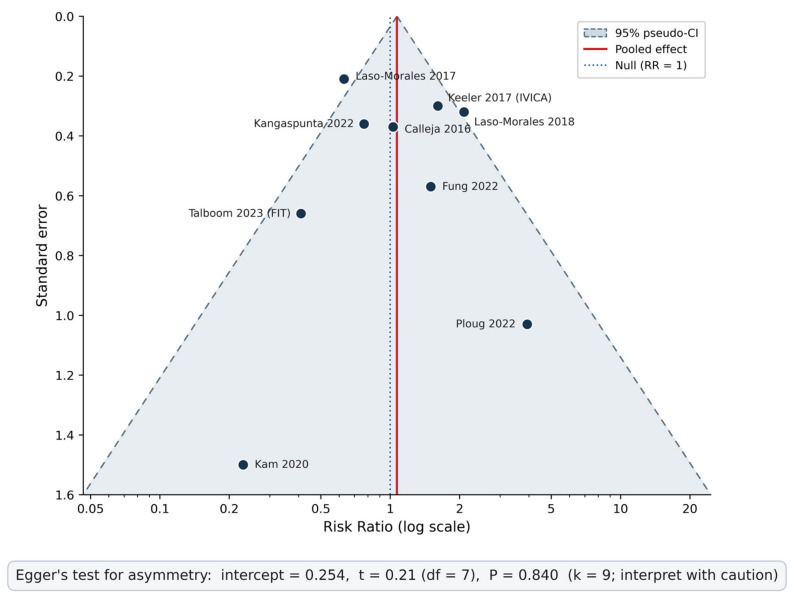
Funnel plot for the primary outcome: infection or infective complications. Funnel plot of study-specific risk ratios for infection or infective complications after parenteral iron versus control in patients with gastrointestinal cancer. The vertical solid line represents the pooled random-effects estimate, and the dotted line represents the null effect (RR = 1). The shaded region indicates the 95% pseudo-confidence limits. Egger’s test did not suggest statistically significant small-study asymmetry (intercept = 0.254; t = 0.21; df = 7; *p* = 0.840); however, this should be interpreted cautiously because only nine studies contributed to the analysis.

**Figure 13:**
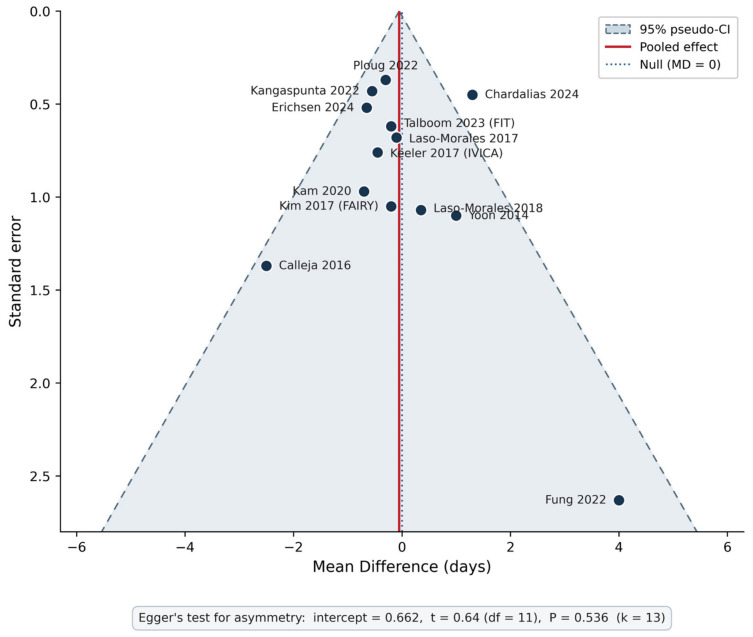
Funnel plot for length of hospital stay. Funnel plot of study-specific mean differences in length of hospital stay after parenteral iron versus control in patients with gastrointestinal cancer. The vertical solid line represents the pooled random-effects mean difference, and the shaded region indicates the 95% pseudo-confidence limits. Egger’s test did not suggest statistically significant small-study asymmetry (intercept = 0.662; t = 0.64; df = 11; *p* = 0.536).

Funnel plots (Figure 12 and Figure 13) were generated for outcomes with sufficient contributing studies. For infectious complications, visual inspection did not suggest clear asymmetry, and Egger’s test was not statistically significant (*p* = 0.840), although interpretation was limited by the small number of studies (k = 9). Similarly, the funnel plot for length of hospital stay showed no clear evidence of asymmetry, supported by a non-significant Egger’s test (*p* = 0.536). Overall, these findings did not suggest major small-study effects, although the publication-bias assessment should be interpreted with caution, given the modest number of included studies and the underlying clinical heterogeneity.

### 3.12. GRADE Summary of Findings

The GRADE approach was used to assess the certainty of evidence for each outcome (high, moderate, low, or very low) (Table 9). Outcomes were evaluated across five domains: risk of bias, inconsistency (heterogeneity), indirectness, imprecision, and publication bias. In accordance with the GRADE Handbook [36], evidence from randomised controlled trials is assigned high certainty, whereas evidence from non-randomised studies is assigned low certainty. Because this review included both randomised and non-randomised evidence, the starting certainty for each outcome was determined by the study design that contributed the greatest statistical weight and by whether sensitivity analyses restricted to randomised trials produced concordant estimates. Downgrading and upgrading decisions were made in accordance with GRADE Handbook guidance [36].

## 4. Discussion

In this systematic review and meta-analysis of 14 comparative studies, parenteral iron was not associated with an increased risk of infection in patients with gastrointestinal cancer. The primary estimate was close to the null and remained stable across sensitivity analyses, excluding imputed events, restricting analyses to randomised trials, analysing non-randomised studies separately, excluding studies at serious risk of bias, and sequentially omitting individual studies. Parenteral iron was also not associated with longer hospital stay, higher hospital readmission, or increased short-term mortality. Overall, these findings provide reassurance that, within the limitations of the available evidence, parenteral iron does not appear to confer a clinically significant excess risk of infection in patients with gastrointestinal cancer [10,11,12,16,17,30,31,32,33].

Anaemia and iron deficiency are highly prevalent in gastrointestinal cancer, particularly among patients undergoing colorectal, gastric and esophagogastric surgery, and are often driven by chronic tumour-related blood loss, inflammation, impaired iron mobilisation and perioperative blood loss [1,2,3,7]. Optimising iron deficiency is therefore a central component of patient blood management, but clinical use of parenteral iron has remained cautious because iron availability may theoretically support bacterial growth and alter host immune responses [6,7,13,14,15]. This concern is particularly relevant in gastrointestinal cancer surgery, where postoperative infectious complications, including surgical site infection, pneumonia, urinary tract infection and intra-abdominal abscess, can prolong recovery and may delay adjuvant oncological treatment [16,17,18,19,30,33]. At the same time, untreated anaemia and allogeneic red blood cell transfusion are themselves associated with adverse postoperative outcomes, including infection [4,5]. In this context, our findings are clinically reassuring. Parenteral iron was not associated with a clinically significant increase in infection risk and was also not associated with longer hospital stay, higher readmission or increased short-term mortality. These results support the perioperative use of parenteral iron as part of anaemia optimisation in appropriately selected patients with gastrointestinal cancer, while recognising that safety should continue to be assessed alongside haematological efficacy.

The direction and magnitude of effect were broadly consistent across the main and sensitivity analyses. The primary infection analysis included both directly reported infection outcomes and imputed infection totals derived from infective subtypes where aggregate infection counts were unavailable. This was necessary because infection reporting varied substantially across the included studies, with some reporting overall infection and others reporting only specific complications such as wound infection, urinary tract infection, pneumonia or intra-abdominal abscess [16,17,30,31,33]. Although such imputation introduces uncertainty, the sensitivity analysis that excluded imputed estimates yielded a very similar pooled effect. This strengthens confidence that the overall conclusion is not driven by the method used to handle incomplete infection reporting. The RCT-only analysis also showed no significant increase in infection risk, although it was limited by the small number of trials and wide confidence intervals [11,12,32].

Although this systematic review focused on infectious outcomes rather than infusion tolerability, clinicians should also recognise that parenteral iron is generally well tolerated in the included gastrointestinal cancer trials, with no treatment-related serious adverse events reported in key studies, but rare transient infusion or hypersensitivity reactions can occur, including abdominal cramping, diaphoresis or cold sweats, abdominal pain and hypotension, and should be managed by pausing or slowing the infusion with appropriate monitoring and supportive care [12,29,37].

The length-of-stay analysis similarly showed no meaningful difference between parenteral iron and control. This is important because even if parenteral iron does not increase infection risk, it could theoretically affect recovery pathways through adverse events, inflammation, or differences in postoperative complications. The pooled mean difference was close to zero, although substantial heterogeneity was observed, likely reflecting differences in cancer site, operative complexity, perioperative pathways, timing of iron administration, iron formulation and reporting methods across the studies contributing length-of-stay data [10,11,12,16,17,28,29,30,31,32,33,34,35]. Therefore, although parenteral iron was not associated with a shorter hospital stay overall, the analysis does not exclude benefit in selected subgroups or specific perioperative protocols.

Readmission and mortality analyses were also reassuring but less definitive. Hospital readmission was reported by only four studies and showed no significant difference between groups [10,12,32,34]. Mortality analyses were event-limited, particularly for 30-day mortality, where very few deaths occurred [17,28,33]. The 90-day mortality point estimate was below unity, but the confidence interval was wide and compatible with no difference [11,17,33,35]. These findings should therefore be interpreted as showing no evidence of harm rather than evidence of a mortality benefit. The limited number of events and variable follow-up windows are consistent with the broader limitations of perioperative anaemia studies in this field. In the exploratory timing subgroup analysis, preoperative parenteral iron was associated with a shorter postoperative length of hospital stay, whereas postoperative iron studies showed the opposite direction of effect, probably reflecting confounding by indication and differences in perioperative pathways. This finding should therefore be interpreted as hypothesis-generating rather than definitive, particularly as a recent RCT-only meta-analysis in colorectal cancer found no significant effect of preoperative intravenous iron on hospital stay despite improvements in haemoglobin and transfusion outcomes [38]; future adequately powered studies should specifically evaluate whether preoperative iron repletion improves recovery-centred outcomes such as length of stay, days alive and out of hospital, postoperative complications and return to intended oncological therapy.

### 4.1. Clinical Importance

The main clinical implication of this review is that parenteral iron appears safe from an infection perspective in patients with gastrointestinal cancer when compared with oral iron, placebo, no iron or standard care. This matters because clinicians often need to optimise anaemia within a short interval between cancer diagnosis and surgery [7]. Oral iron may be poorly tolerated, slowly effective and less reliable in the presence of inflammation or impaired gastrointestinal absorption [8,9]. Parenteral iron offers a pragmatic route to replenish iron stores more rapidly, particularly in patients with moderate anaemia, iron deficiency, poor oral tolerance or an urgent surgical timeline [7,10,11,12].

Perioperative recovery is also influenced by patient-level and surgical-pathway factors that were not consistently extractable from the included studies. Nutritional status and early dietitian-led optimisation are important components of supportive cancer care, particularly in patients undergoing gastrointestinal surgery [39]. Similarly, operative access and intraoperative perfusion assessment may influence blood loss, anastomotic safety and downstream inflammatory or infective complications; minimally invasive colorectal approaches and indocyanine green fluorescence techniques have been used to support vascular identification and perfusion assessment, although these variables were not consistently reported in the studies included in this review [40].

These findings support the continued perioperative use of parenteral iron within patient blood management pathways for appropriately selected patients with gastrointestinal cancer [6,7]. The results are especially relevant for colorectal cancer surgery, where most of the included evidence is concentrated [10,11,12,16,17,30,31,32,33,34,35]. Within the included dataset, evidence for upper gastrointestinal disease was limited to gastric cancer studies, and no extractable oesophageal, pancreatic, or hepatobiliary comparative outcomes were available for the meta-analysis [27,28,29].

The review also highlights the need for better outcome reporting in future studies. Infection was not consistently reported as a standardised, patient-level outcome. Some studies reported only total complications, others reported selected infection subtypes, and some reported adverse events without clear infective classification [16,17,30,31,33]. Future trials should report infections using standard definitions and should provide arm-level counts for overall infection, surgical site infection, pneumonia, urinary tract infection, intra-abdominal abscess, and sepsis. This would improve the reliability of future evidence synthesis and reduce reliance on imputed outcomes, in keeping with PRISMA and Cochrane reporting principles [20,21].

### 4.2. Limitations

This review has several limitations. First, the evidence base was predominantly observational. Although four randomised trials were included overall, only three contributed to the primary infection analysis [11,12,29,32]. Many observational studies were susceptible to confounding by indication because patients selected for parenteral iron often differed from controls in baseline haemoglobin, severity of iron deficiency, surgical timing, comorbidity, or clinical pathway [10,16,17,27,28,30,31,33,34,35]. The risk-of-bias assessment reflected these limitations, with several non-randomised studies judged to be at serious risk of bias according to the ROBINS-I [23].

Second, infection definitions varied across studies. Some studies reported overall infective complications, whereas others reported individual events such as wound infection, pneumonia, urinary tract infection or abscess [16,17,30,31,33]. In several studies, total infection counts had to be imputed from reported subtypes or percentages. Although sensitivity analyses suggested that these imputations did not materially affect the pooled result, the possibility of double-counting or undercounting cannot be ruled out.

Third, there was clinical heterogeneity across studies. Included populations varied by cancer site, operative approach, timing of iron administration, iron formulation, dose, and comparator type [10,11,12,16,17,27,28,29,30,31,32,33,34,35]. Some studies evaluated preoperative iron, others postoperative iron, and some were embedded within broader anaemia management pathways. These differences may influence infection risk and other perioperative outcomes. Data on baseline nutritional status, formal nutrition support, surgical access, indocyanine green use, and intraoperative wound or operative-field contamination were not reported consistently enough for meta-analysis.

Fourth, several secondary outcomes were inconsistently reported. Length of stay was available for most studies, but some studies reported medians and interquartile ranges rather than means and standard deviations, requiring statistical conversion using established methods [22]. Readmission and mortality were reported by fewer studies, and mortality analyses were limited by low event rates. The available evidence was therefore insufficient to reliably detect small differences in mortality or rare safety outcomes.

Finally, most included studies focused on colorectal cancer, limiting generalisability to other gastrointestinal malignancies [10,11,12,16,17,30,31,32,33,34,35]. Evidence in gastric, oesophageal, pancreatic and hepatobiliary cancers remains less developed, with only a small number of gastric cancer studies contributing extractable outcomes [27,28,29]. Future adequately powered studies should evaluate parenteral iron across broader gastrointestinal cancer populations and should incorporate standardised safety, recovery and patient-centred outcomes.

## 5. Conclusions

In patients with gastrointestinal cancer, parenteral iron was not associated with an increased risk of infection compared with oral iron, placebo, no iron or standard care. It was also not associated with prolonged hospital stay, higher readmission rates, increased short-term mortality, or surgical site/wound infection. These findings provide reassuring evidence regarding the perioperative safety of parenteral iron as part of anaemia optimisation in gastrointestinal cancer, particularly in colorectal cancer surgery. However, confidence in the evidence is limited by the predominance of observational studies, heterogeneous definitions of infection, and incomplete outcome reporting. Future trials should use standardised infection definitions, report patient-level, arm-specific safety outcomes, and evaluate whether parenteral iron improves clinically important outcomes beyond haemoglobin correction, including quality of life.

## Figures and Tables

**Table 1:** Characteristics of included comparative studies.

Study	Design	Cancer Site	Timing	Iron Formulation	Comparator
Jeong 2014 [27]	Observational	Gastric	Postoperative	Iron sucrose	Observation/no treatment
Yoon 2014 [28]	Observational	Gastric	Postoperative	Iron sucrose	Observation/no treatment
Calleja 2016 [10]	Observational	Colon	Preoperative	Ferric carboxymaltose	Oral/no IV iron historical controls
Keeler 2017 (IVICA) [11]	RCT	Colorectal	Preoperative	Ferric carboxymaltose	Oral iron
Kim 2017 (FAIRY) [29]	RCT	Gastric	Postoperative	Ferric carboxymaltose	Placebo
Laso-Morales 2017 [16]	Observational	Colorectal	Preoperative	Iron sucrose or ferric carboxymaltose	Oral iron or no iron
Laso-Morales 2018 [30]	Observational	Colorectal	Postoperative	Iron sucrose	Oral iron or no iron
Kam 2020 [31]	Propensity score matched cohort	Colorectal	Preoperative	Iron sucrose or ferric derisomaltose	Historical standard care
Fung 2022 [32]	Pilot RCT	Colorectal	Preoperative	Ferric derisomaltose/isomaltoside	Usual care/no iron
Kangaspunta 2022 [33]	Observational	Colon	Preoperative	Ferric carboxymaltose	No IV iron/standard care
Ploug 2022 [17]	Registry cohort	Colorectal	Preoperative	Ferric derisomaltose/isomaltoside	No iron therapy
Talboom 2023 (FIT) [12]	RCT	Colorectal	Preoperative	Ferric carboxymaltose	Oral iron
Chardalias 2024 [34]	Prospective observational	Colorectal	Postoperative	Ferric carboxymaltose	Routine care/no iron
Erichsen 2024 [35]	Observational	Colorectal	Preoperative	IV iron screening pathway	No IV iron/usual care

**Table 2:** Sensitivity and subgroup analyses for infection/infective complications.

Analysis	Studies	Events/N, Iron	Events/N, Control	Pooled RR (95% CI)	I^2^	*p* Value	Interpretation
Primary infection analysis	9	126/899	104/768	1.07 (0.71–1.62)	57%	0.754	No increased infection risk
Directly reported infection events only	5	82/523	53/321	1.04 (0.55–1.98)	64%	0.906	Robust after excluding imputed estimates
Randomised controlled trials only	3	30/169	26/183	1.17 (0.56–2.43)	44%	0.671	No significant increase in RCTs
Non-randomised studies only	6	96/730	78/585	1.03 (0.60–1.75)	62%	0.920	Consistent with primary analysis
Excluding non-randomised studies at serious risk of bias	5	40/329	30/293	1.21 (0.61–2.40)	34%	0.583	No signal of harm after excluding serious-risk observational studies

**Table 3:** Leave-one-out sensitivity analysis for the primary infection outcome.

Study Omitted	Studies Remaining	Pooled RR (95% CI)	I^2^	*p* Value
Keeler 2017 [11] (IVICA)	8	0.99 (0.63–1.56)	55%	0.954
Talboom 2023 [12] (FIT)	8	1.15 (0.75–1.75)	58%	0.526
Fung 2022 [32]	8	1.03 (0.66–1.62)	61%	0.888
Calleja 2016 [10]	8	1.07 (0.66–1.75)	62%	0.774
Laso-Morales 2017 [16]	8	1.23 (0.83–1.83)	37%	0.313
Laso-Morales 2018 [30]	8	0.94 (0.63–1.40)	43%	0.764
Kam 2020 [31]	8	1.10 (0.72–1.67)	60%	0.658
Kangaspunta 2022 [33]	8	1.13 (0.70–1.81)	61%	0.623
Ploug 2022 [17]	8	1.02 (0.67–1.54)	58%	0.927

**Table 4:** Length-of-stay sensitivity analysis excluding converted median/IQR studies.

Analysis	Studies Included	Effect Measure	Pooled Effect (95% CI)	I^2^	*p* Value	Interpretation
Primary length-of-stay analysis	13	Mean difference, days	−0.05 (−0.66 to 0.57)	65%	0.885	No meaningful difference
Direct mean ± SD studies only	5	Mean difference, days	0.25 (−1.25 to 1.75)	66%	0.742	Consistent after excluding converted values

**Table 5:** Summary of meta-analysis, subgroup and sensitivity analyses.

Outcome	Studies (k)	Pooled Effect (95% CI)	I^2^	*p* Value
Infection/infective complications	9	RR 1.07 (0.71 to 1.62)	57%	0.754
Infection: imputed studies excluded	5	RR 1.04 (0.55 to 1.98)	64%	0.906
Infection: randomised trials only	3	RR 1.17 (0.56 to 2.43)	44%	0.671
Infection: non-randomised studies only	6	RR 1.03 (0.60 to 1.75)	62%	0.920
Infection: serious risk-of-bias studies excluded	5	RR 1.21 (0.61 to 2.40)	34%	0.583
Length of hospital stay	13	MD −0.05 days (−0.66 to 0.57)	65%	0.885
Length of stay: direct mean ± SD only	5	MD 0.25 days (−1.25 to 1.75)	66%	0.742
Hospital readmission	4	RR 0.76 (0.35 to 1.64)	0%	0.480
30-day all-cause mortality	3	RR 0.49 (0.09 to 2.61)	0%	0.406
90-day all-cause mortality	4	RR 0.69 (0.35 to 1.36)	0%	0.286
Surgical site/wound infection	4	RR 0.81 (0.40 to 1.65)	11%	0.561

**Table 6:** Subgroup analysis by cancer site.

Outcome and Subgroup	k	Participants (Iron/Control)	Pooled Effect (95% CI)	I^2^	*p* Value	Interpretation
**Primary outcome: infection/infective complications**						
Colorectal/colon cancer (only contributing subgroup)	9	899/768	RR 1.07 (0.71 to 1.62)	57%	0.754	Primary infection analysis (k = 9). All contributing studies were colorectal or colon cancer; no included gastric cancer study reported extractable infection counts.
Gastric cancer	0	—	Not estimable	—	—	No included gastric cancer study (Jeong 2014 [27], Yoon 2014 [28], Kim 2017 [29] FAIRY) reported patient-level infection counts in a form that could be pooled.
**Secondary outcome: length of hospital stay**						
Colorectal/colon cancer	11	991/908	MD −0.21 days (−0.81 to +0.40)	63%	0.500	Pooled mean difference essentially null; consistent with the full 13-study analysis.
Gastric cancer	2	291/286	MD +1.33 days (−1.71 to +4.37)	75%	0.391	Two-study estimate (Kim 2017 [29] FAIRY, Yoon 2014 [28]) with substantial heterogeneity and wide confidence interval; interpret with caution.
**Secondary outcome: hospital readmission**						
Colorectal/colon cancer (only contributing subgroup)	4	263/323	RR 0.76 (0.35 to 1.64)	0%	0.480	Primary readmission analysis. No included gastric cancer study reported readmission counts.
Gastric cancer	0	—	Not estimable	—	—	No gastric contributor.
**Secondary outcome: 30-day all-cause mortality**						
Colorectal/colon cancer	2	302/186	See note	—	—	Kangaspunta 2022 [33] (1/180 vs. 3/138) and Ploug 2022 [17] (1/122 vs. 0/48). The two-study estimate adds little to the primary analysis and is not reported separately to avoid overinterpretation.
Gastric cancer	1	63/60	See note	—	—	Yoon 2014 [28] only (0/63 vs. 0/60); zero events in both arms preclude meaningful subgroup estimation.
**Secondary outcome: 90-day all-cause mortality**						
Colorectal/colon cancer (only contributing subgroup)	4	411/341	RR 0.69 (0.35 to 1.36)	0%	0.286	Primary 90-day mortality analysis. No included gastric cancer study reported 90-day mortality.
Gastric cancer	0	—	Not estimable	—	—	No gastric contributor.

RR = risk ratio; MD = mean difference; CI = confidence interval; I^2^ = proportion of variability across studies attributable to heterogeneity rather than chance. Pooled estimates use the DerSimonian–Laird random-effects model on the natural-log scale for ratio outcomes and on the original scale for mean differences. Subgroups with k < 2 contributing studies are reported descriptively only.

**Table 7:** Subgroup analysis by iron formulation.

Outcome and Formulation Subgroup	k	Participants (Iron/Control)	Pooled Effect (95% CI)	I^2^	*p* Value	Studies Contributing
**Primary outcome: infection/infective complications**						
Ferric carboxymaltose	4	440/456	RR 1.00 (0.63 to 1.61)	40%	0.993	Keeler 2017 [11] (IVICA), Talboom 2023 [12] (FIT), Calleja 2016 [10], Kangaspunta 2022 [33]
Other formulations (iron sucrose, ferric derisomaltose/iron isomaltoside, or mixed)	5	459/312	RR 1.23 (0.56 to 2.74)	70%	0.606	Fung 2022 [32] (ferric derisomaltose), Laso-Morales 2017 [16] (iron sucrose/ferric carboxymaltose mixed), Laso-Morales 2018 [30] (iron sucrose), Kam 2020 [31] (iron sucrose/ferric derisomaltose mixed), Ploug 2022 [17] (ferric derisomaltose)
Test for subgroup difference	—	—	Q_between = 0.10, df = 1	—	0.752	No evidence that infection risk differed by iron formulation.
**Secondary outcome: length of hospital stay**						
Ferric carboxymaltose	6	706/728	MD −0.38 days (−1.41 to +0.65)	76%	0.471	Kim 2017 [29] (FAIRY), Keeler 2017 [11] (IVICA), Talboom 2023 [12] (FIT), Calleja 2016 [10], Kangaspunta 2022 [33], Chardalias 2024 [34]
Other formulations	7	576/466	MD +0.21 days (−0.61 to +1.04)	55%	0.612	Yoon 2014 [28] (iron sucrose), Laso-Morales 2017 [16], Laso-Morales 2018 [30] (iron sucrose), Fung 2022 [32] (ferric derisomaltose), Kam 2020 [31], Ploug 2022 [17] (ferric derisomaltose), Erichsen 2024 [35] (mixed pathway)
Test for subgroup difference	—	—	Q_between = 0.48, df = 1	—	0.488	No evidence that length-of-stay effect differed by iron formulation.

RR = risk ratio; MD = mean difference; CI = confidence interval; I^2^ = proportion of variability across studies attributable to heterogeneity rather than chance. Q_between = Cochran chi-squared statistic for between-subgroup heterogeneity, computed as Q_total − Σ Q_within from DerSimonian–Laird random-effects fits within each subgroup. Pooled estimates use the DerSimonian–Laird random-effects model. Ferric derisomaltose is also known as iron isomaltoside; the two terms are used interchangeably in the source literature. Studies are categorised as ferric carboxymaltose only when this was the single formulation administered to all participants in the parenteral iron arm; cohorts in which more than one formulation was used (Laso-Morales 2017 [16] and Kam 2020 [31]) are classified as “other formulations” for the conservative subgroup comparison. The Q-between test has limited statistical power when within-subgroup heterogeneity is substantial (I^2^ > 50%), as is the case here for one or both subgroups; non-significant Q-between values should therefore be interpreted as the absence of demonstrable subgroup differences rather than as evidence of equivalence.

**Table 8:** Subgroup analysis by timing of parenteral iron administration.

Outcome and Timing Subgroup	k	Participants (Iron/Control)	Pooled Effect (95% CI)	I^2^	*p* Value	Studies Contributing
**Primary outcome: infection/infective complications**						
Preoperative iron	8	852/676	RR 0.94 (0.63 to 1.40)	43%	0.764	Keeler 2017 [11] (IVICA), Talboom 2023 [12] (FIT), Fung 2022 [32], Calleja 2016 [10], Laso-Morales 2017 [30], Kam 2020 [31], Kangaspunta 2022 [33], Ploug 2022 [17]
Postoperative iron	1	47/92	Not pooled—descriptive	—	—	Laso-Morales 2018 [30] only: study-level RR 2.09 (1.13 to 3.84) for postoperative iron sucrose in patients with severe postoperative anaemia. Single-study estimate; not separately pooled.
Test for subgroup difference	—	—	Not performed	—	—	Not performed because the postoperative subgroup contained only one study.
**Secondary outcome: length of hospital stay**						
Preoperative iron	9	908/774	MD −0.51 days (−0.98 to −0.05)	30%	0.031	Calleja 2016 [10], Laso-Morales 2017 [16], Keeler 2017 [11] (IVICA), Talboom 2023 [12] (FIT), Fung 2022 [32], Kam 2020 [31], Kangaspunta 2022 [33], Ploug 2022 [17], Erichsen 2024 [35]
Postoperative iron	4	374/420	MD +1.26 days (+0.30 to +2.23)	28%	0.010	Kim 2017 [29] (FAIRY), Yoon 2014 [28], Laso-Morales 2018 [30], Chardalias 2024 [34]
Test for subgroup difference	—	—	Q_between = 19.12, df = 1	—	<0.001	Strong evidence of a difference in length-of-stay effect by timing of iron administration. Preoperative iron is associated with shorter postoperative length of stay; postoperative iron studies show longer length of stay in iron-treated patients, almost certainly reflecting confounding by indication (postoperative iron is given to patients with more severe perioperative anaemia, who also have longer admissions).

RR = risk ratio; MD = mean difference; CI = confidence interval; I^2^ = proportion of variability across studies attributable to heterogeneity rather than chance. Q_between = Cochran chi-squared statistic for between-subgroup heterogeneity, computed as Q_total − Σ Q_within from DerSimonian–Laird random-effects fits. Pooled estimates use the DerSimonian–Laird random-effects model.

**Table 9:** GRADEpro summary of findings.

Outcome	Studies (k)	Participants	Relative Effect (95% CI)	Anticipated Absolute Effects	RoB	Inc	Ind	Imp	Pub	Certainty
**Infection/infective complications**	9 (3 RCTs, 6 OBS)	1667	RR 1.07 (0.71 to 1.62)	Control: 135/1000 Iron: 145/1000 (96 to 219)	+	+	0	0	0	**LOW** ⊕⊕⊖⊖
**Infection: RCTs only**	3 RCTs	352	RR 1.17 (0.56 to 2.43)	Control: 142/1000 Iron: 166/1000 (80 to 345)	0	0	0	+	NA	**MODERATE** ⊕⊕⊕⊖
**Length of hospital stay**	13 (4 RCTs, 9 OBS)	≈2476	MD −0.05 days (−0.66 to 0.57)	Control: Median LOS ≈7–9 d Iron: Same as control	0	+	0	0	0	**MODERATE** ⊕⊕⊕⊖
**Hospital readmission**	4 (2 RCTs, 2 OBS)	586	RR 0.76 (0.35 to 1.64)	Control: 50/1000 Iron: 38/1000 (17 to 82)	+	0	0	+	NA	**LOW** ⊕⊕⊖⊖
**30-day all-cause mortality**	3 OBS	611	RR 0.49 (0.09 to 2.61)	Control: 12/1000 Iron: 6/1000 (1 to 31)	+	0	0	++	NA	**VERY LOW** ⊕⊖⊖⊖
**90-day all-cause mortality**	4 (1 RCT, 3 OBS)	752	RR 0.69 (0.35 to 1.36)	Control: 62/1000 Iron: 42/1000 (22 to 84)	+	0	0	+	NA	**LOW** ⊕⊕⊖⊖
**Surgical site/wound infection**	4 OBS	879	RR 0.81 (0.40 to 1.65)	Control: 45/1000 Iron: 36/1000 (18 to 73)	++	0	0	+	NA	**VERY LOW** ⊕⊖⊖⊖

RR = risk ratio; MD = mean difference; CI = confidence interval; RoB = risk of bias; Inc = inconsistency; Ind = indirectness; Imp = imprecision; Pub = publication bias. Domain symbols: 0 = no serious concern; + = serious (downgrade one level); ++ = very serious (downgrade two levels); NA = not assessed (k < 10, too few studies for funnel-plot or Egger test). Anticipated absolute effects assume the pooled control risk; the absolute risk of parenteral iron is calculated by applying the pooled relative effect to that control risk.

## Data Availability

All extracted data are presented in the main text and Appendix A. The full extraction spreadsheets are available from the corresponding author on reasonable request.

## Supplementary Materials

The following supporting information can be downloaded at: https://www.mdpi.com/article/10.3390/cancers18121859/s1, Table S1: PRISMA 2020 Checklist.

## Appendix A. Database-Specific Search Strategies

Appendix A contains the final exported line-by-line search strategies for MEDLINE, Embase, and Scopus, including the date searched (20 February 2026), the database platform, controlled vocabulary terms, free-text terms, and search yields.

Database: Ovid MEDLINE(R) ALL <1946 to 20 February 2026>
Search Strategy:

1. exp Ferric Compounds/(45389)
2. exp Ferrous Compounds/(19569)
3. exp Iron/(117820)
4. (alvofer or colliron or faremio or ferion or feriv or fermed or ferri saccharate or ferric hydroxide sucrose or ferric oxide saccharate or ferric oxide,saccharated or ferric saccharate or ferrinemia or ferrisaccharate or ferrivenin or ferrologic or ferrous saccharate or ferrovin or fesin or hemafer s or hemafer-s or idafer or (iron adj2 hydroxide sucrose complex) or iron saccharate or iron sucrose or ironcrose or iviron or nefro-fer or nefrofer or neo ferrum or nephroferol or proferrin or referen or reoxyl or saccharate ferric or saccharate iron or saccharated ferric oxide or saccharated iron oxide or sucro fer or sucrofer or sucroven or veniron or venofer or venotrix).tw,kf. (1056)
5. (ferric carboxymaltose or Ferinject or Injectafer or Iroprem).tw,kf. (986)
6. (ferlecit or ferlixit or ferric gluconate or ferrigluconate or ferrlecit or gluconate ferric sodium or (iron adj2 gluconate) or sodium ferrigluconate or intravenous iron sucrose or iron sucrose injection* or venofer).tw,kf. (647)
7. (diafer or ferric derisomaltose or iron isomaltoside or monofer or monafer or monoferro or monover or ferumoxytol or feraheme or rienso).tw,kf. (938)
8. (IV iron or “IV iron” or iron therapy or ((intravenous* or inject* or infus* or parenteral) adj3 iron)).tw,kf. (8098)
9. (ferumoxytol or injectafer or infed or cosmofer or iron polymaltose or triferic or ferric pyrophosphate citrate).mp. (841)
10. (anaemex or cosmofer or dexferrum or dexiron or dextrafer or dextran fe or dextran ferrous or dextran iron or driken or fenate or fer dextran or ferric dextran or ferridex or tranferrisat or ferrodex or ferrodextran or ferrous dextran or ferrum lek or fervetag or hibiron or imferdex or imferon or impheron or imposil or infed or infufer or iron dextran complex or ironate or monofar or proferdex or uniferon or uniferon or uniferron).mp. (1602)
11. 1 or 2 or 3 or 4 or 5 or 6 or 7 or 8 or 9 or 10 (169577)
12. exp Administration, Intravenous/(151852)
13. (intravenous* or IV or “IV” or infus* or inject* or parenteral*).tw,kf. (1913367)
14. 12 or 13 (1948894)
15. 11 and 14 (19275)
16. (Gastrointestinal or GI tract or oesophagus or gastric canal or stomach or HPB or hepatopancreatobiliary or hepatic or liver or biliary tract or colon or GB cancer or gallbladder cancer or small bowel or rectal or colorectal or cholangiocarcinoma or pancreatic cancer or bowel cancer or GI).mp. [mp=title, book title, abstract, original title, name of substance word, subject heading word, floating sub-heading word, keyword heading word, organism supplementary concept word, protocol supplementary concept word, rare disease supplementary concept word, unique identifier, synonyms, population supplementary concept word, anatomy supplementary concept word] (2721275)
17. Gastrointestinal.mp. or Gastrointestinal Diseases/or Gastrointestinal Stromal Tumors/or exp Gastrointestinal Neoplasms/(953796)
18. exp Colorectal Neoplasms/or exp Intestinal Neoplasms/or exp Colonic Neoplasms/or bowel cancer.mp. (292581)
19. exp Pancreatic Neoplasms/(101588)
20. (cancer or chemotherapy or malignancy or tumor or tumour or radiotherapy or targeted therapy or neoplasm).mp. [mp=title, book title, abstract, original title, name of substance word, subject heading word, floating sub-heading word, keyword heading word, organism supplementary concept word, protocol supplementary concept word, rare disease supplementary concept word, unique identifier, synonyms, population supplementary concept word, anatomy supplementary concept word] (4574065)
21. exp Stomach Neoplasms/or oesophagus cancer.mp. or exp Esophageal Neoplasms/(177392)
22. 16 or 17 or 18 or 19 or 21 (2844745)
23. 20 and 22 (955330)
24. 15 and 23 (621)

Database: Embase <1974 to 2026 Week 08>
Search Strategy:

1. exp Ferric Compounds/(26807)
2. exp ferrous ion/(22450)
3. exp Ferrous Compounds/(22450)
4. exp Iron/(233856)
5. (alvofer or colliron or faremio or ferion or feriv or fermed or ferri saccharate or ferric hydroxide sucrose or ferric oxide saccharate or ferric oxide,saccharated or ferric saccharate or ferrinemia or ferrisaccharate or ferrivenin or ferrologic or ferrous saccharate or ferrovin or fesin or hemafer s or hemafer-s or idafer or (iron adj2 hydroxide sucrose complex) or iron saccharate or iron sucrose or ironcrose or iviron or nefro-fer or nefrofer or neo ferrum or nephroferol or proferrin or referen or reoxyl or saccharate ferric or saccharate iron or saccharated ferric oxide or saccharated iron oxide or sucro fer or sucrofer or sucroven or veniron or venofer or venotrix).tw,kf. (2479)
6. (ferric carboxymaltose or Ferinject or Injectafer or Iroprem).tw,kf. (2372)
7. (ferric carboxymaltose or Ferinject or Injectafer or Iroprem).mp. [mp=title, abstract, heading word, drug trade name, original title, device manufacturer, drug manufacturer, device trade name, keyword heading word, floating subheading word, candidate term word] (3206)
8. (ferlecit or ferlixit or ferric gluconate or ferrigluconate or ferrlecit or gluconate ferric sodium or (iron adj2 gluconate) or sodium ferrigluconate or intravenous iron sucrose or iron sucrose injection* or venofer).tw,kf. (1826)
9. (ferlecit or ferlixit or ferric gluconate or ferrigluconate or ferrlecit or gluconate ferric sodium or (iron adj2 gluconate) or sodium ferrigluconate or intravenous iron sucrose or iron sucrose injection* or venofer).mp. [mp=title, abstract, heading word, drug trade name, original title, device manufacturer, drug manufacturer, device trade name, keyword heading word, floating subheading word, candidate term word] (2165)
10. (ferlecit or ferlixit or ferric gluconate or ferrigluconate or ferrlecit or gluconate ferric sodium or (iron adj2 gluconate) or sodium ferrigluconate or intravenous iron sucrose or iron sucrose injection* or venofer).tw,kf. (1826)
11. (diafer or ferric derisomaltose or iron isomaltoside or monofer or monafer or monoferro or monover or ferumoxytol or feraheme or rienso).tw,kf. (2194)
12. (diafer or ferric derisomaltose or iron isomaltoside or monofer or monafer or monoferro or monover or ferumoxytol or feraheme or rienso).mp. [mp=title, abstract, heading word, drug trade name, original title, device manufacturer, drug manufacturer, device trade name, keyword heading word, floating subheading word, candidate term word] (2916)
13. (IV iron or “IV iron” or iron therapy or ((intravenous* or inject* or infus* or parenteral) adj3 iron)).tw,kf. (13710)
14. (ferumoxytol or injectafer or infed or cosmofer or iron polymaltose or triferic or ferric pyrophosphate citrate).mp. (3139)
15. (anaemex or cosmofer or dexferrum or dexiron or dextrafer or dextran fe or dextran ferrous or dextran iron or driken or fenate or fer dextran or ferric dextran or ferridex or tranferrisat or ferrodex or ferrodextran or ferrous dextran or ferrum lek or fervetag or hibiron or imferdex or imferon or impheron or imposil or infed or infufer or iron dextran complex or ironate or monofar or proferdex or uniferon or uniferon or uniferron).mp. [mp=title, abstract, heading word, drug trade name, original title, device manufacturer, drug manufacturer, device trade name, keyword heading word, floating subheading word, candidate term word] (993)
16. (anaemex or cosmofer or dexferrum or dexiron or dextrafer or dextran fe or dextran ferrous or dextran iron or driken or fenate or fer dextran or ferric dextran or ferridex or tranferrisat or ferrodex or ferrodextran or ferrous dextran or ferrum lek or fervetag or hibiron or imferdex or imferon or impheron or imposil or infed or infufer or iron dextran complex or ironate or monofar or proferdex or uniferon or uniferon or uniferron).mp. (993)
17. 1 or 2 or 3 or 4 or 5 or 6 or 7 or 8 or 9 or 10 or 11 or 12 or 13 or 14 or 15 or 16 (274512)
18. exp Administration, Intravenous/(467694)
19. exp intravenous drug administration/(467694)
20. Administration, Intravenous.mp. [mp=title, abstract, heading word, drug trade name, original title, device manufacturer, drug manufacturer, device trade name, keyword heading word, floating subheading word, candidate term word] (634)
21. (intravenous* or IV or “IV” or infus* or inject* or parenteral*).tw,kf. (2792524)
22. 18 or 19 or 20 or 21 (3013506)
23. 17 and 22 (27354)
24. (Gastrointestinal or GI tract or oesophagus or gastric canal or stomach or HPB or hepatopancreatobiliary or hepatic or liver or biliary tract or colon or GB cancer or gallbladder cancer or small bowel or rectal or colorectal or cholangiocarcinoma or pancreatic cancer or bowel cancer or GI).mp. [mp=title, abstract, heading word, drug trade name, original title, device manufacturer, drug manufacturer, device trade name, keyword heading word, floating subheading word, candidate term word] (4173804)
25. Gastrointestinal/or Gastrointestinal Diseases/or Gastrointestinal Stromal Tumo?rs/or exp Gastrointestinal Neoplasms/(924364)
26. Colorectal Neoplasms/or exp Intestinal Neoplasms/or exp Colonic Neoplasms/or bowel cancer.mp. [mp=title, abstract, heading word, drug trade name, original title, device manufacturer, drug manufacturer, device trade name, keyword heading word, floating subheading word, candidate term word] (613163)
27. exp Colorectal Neoplasms/or exp Intestinal Neoplasms/or exp Colonic Neoplasms/or bowel cancer.mp. (613163)
28. exp Pancreatic Neoplasms/(232441)
29. exp Stomach Neoplasms/or ?esophagus cancer.mp. or exp ?Esophageal Neoplasms/[mp=title, abstract, heading word, drug trade name, original title, device manufacturer, drug manufacturer, device trade name, keyword heading word, floating subheading word, candidate term word] (318756)
30. 24 or 25 or 26 or 27 or 28 or 29 (4354085)
31. (cancer or chemotherapy or malignancy or tumo?r or tumour or radiotherapy or targeted therapy or neoplasm).mp. [mp=title, abstract, heading word, drug trade name, original title, device manufacturer, drug manufacturer, device trade name, keyword heading word, floating subheading word, candidate term word] (7413441)
32. 30 and 31 (1869362)
33. 23 and 32 (1704)

**Database:** Scopus
**Platform:** Elsevier Scopus
**Date searched:** 20 February 2026
**Search yield:** 200 records
**Scopus search string**
TITLE-ABS-KEY (
(
“intravenous iron” OR “IV iron” OR “parenteral iron” OR
“iron infusion” OR “iron injection” OR
“iron therapy” OR
“ferric carboxymaltose” OR ferinject OR injectafer OR iroprem OR
“iron sucrose” OR venofer OR “ferric hydroxide sucrose” OR
“saccharated iron oxide” OR “iron saccharate” OR
“ferric gluconate” OR “sodium ferric gluconate” OR ferrlecit OR ferlecit OR
“ferric derisomaltose” OR “iron isomaltoside” OR monofer OR monoferric OR
“iron polymaltose” OR
ferumoxytol OR feraheme OR rienso OR
“iron dextran” OR cosmofer OR infed OR dexferrum OR
“ferric pyrophosphate citrate” OR triferic
)
AND
(
“gastrointestinal cancer” OR “gastrointestinal neoplasm*” OR
“gastrointestinal malignan*” OR
“GI cancer” OR “GI malignan*” OR
“colorectal cancer” OR “colorectal neoplasm*” OR
“colon cancer” OR “colonic neoplasm*” OR
“rectal cancer” OR “rectal neoplasm*” OR
“bowel cancer” OR
“gastric cancer” OR “stomach cancer” OR “stomach neoplasm*” OR
“oesophageal cancer” OR “esophageal cancer” OR
“oesophagogastric cancer” OR “esophagogastric cancer” OR
“gastro-oesophageal cancer” OR “gastroesophageal cancer” OR
“pancreatic cancer” OR “pancreatic neoplasm*” OR
“hepatobiliary cancer” OR “hepatobiliary neoplasm*” OR
“biliary tract cancer” OR “biliary tract neoplasm*” OR
cholangiocarcinoma OR
“gallbladder cancer” OR “gallbladder neoplasm*” OR
“small bowel cancer” OR “small intestinal cancer” OR
“anal cancer”
)
)
