# Supplementary material for: Does Parenteral Iron Increase the Risk of Infection in Patients with Gastrointestinal Cancer? A Systematic Review and Meta-Analysis
Source: Cancers (Basel). 2026 Jun 6;18(12):1859. doi: 10.3390/cancers18121859 (PMC13297121; doi:10.3390/cancers18121859)
Supplement: Supplementary file 1 [file cancers-18-01859-s001.zip › cancers-4348869-supplementary.pdf]

Table S1: PRISMA 2020 Checklist

Section and Topic	Item	Checklist item	Location where item is reported
**TITLE**			
Title	1	Identify the report as a systematic review.	Title page: "Does Parenteral Iron Increase the Risk of Infection in Patients with Gastrointestinal Cancer? A Systematic Review and Meta-analysis".
**ABSTRACT**			
Abstract	2	See the PRISMA 2020 for Abstracts checklist.	Abstract: Background, Methods, Results and Conclusion.
**INTRODUCTION**			
Rationale	3	Describe the rationale for the review in the context of existing knowledge.	Introduction: paragraphs describing anaemia, iron deficiency, parenteral iron, and infection concerns in gastrointestinal cancer.
Objectives	4	Provide an explicit statement of the objective(s) or question(s) the review addresses.	Introduction: final paragraph stating the primary and secondary objectives.
**METHODS**			
Eligibility criteria	5	Specify the inclusion and exclusion criteria for the review and how studies were grouped for the syntheses.	Methods: Eligibility criteria; Outcomes; Statistical analysis. Study grouping is described by design, comparator, cancer site, iron formulation, timing of administration and outcome-specific eligibility.
Information sources	6	Specify all databases, registers, websites, organisations, reference lists and other sources searched or consulted to identify studies. Specify the date when each source was last searched or consulted.	Methods: Search Strategy and Information Sources; Results: Study Selection; Appendix A. Databases searched: Embase, MEDLINE, CENTRAL and Scopus, from inception to 20 February 2026, with citation searching.
Search strategy	7	Present the full search strategies for all databases, registers and websites, including any filters and limits used.	Appendix A: database-specific search strategies for MEDLINE, Embase and Scopus, including date searched, platform, search terms and yields. CENTRAL is listed among the searched databases in the Methods and Results; if the CENTRAL line-by-line strategy is not included in Appendix A, it should be provided as a separate supplementary search appendix.
Selection process	8	Specify the methods used to decide whether a study met the inclusion criteria of the review, including how many reviewers screened each record and each report retrieved, whether they worked independently, and if applicable, details of automation tools used in the process.	Methods: Search strategy and information sources. Titles/abstracts and full texts were screened independently by two reviewers, with disagreements resolved by discussion or a third reviewer.
Data collection process	9	Specify the methods used to collect data from reports, including how many reviewers collected data from each report, whether they worked independently, any processes for obtaining or confirming data from study investigators, and if applicable, details of automation tools used in the process.	Methods: Search strategy and information sources; Data extraction and risk of bias. Data extraction was performed independently by two reviewers using prespecified forms; discrepancies were resolved by consensus.
Data items	10a	List and define all outcomes for which data were sought. Specify whether all results that were compatible with each outcome domain in each study were sought (e.g. for all measures, time points, analyses), and if not, the methods used to decide which results to collect.	Methods: Outcomes. Primary outcome: infection/infective complications. Secondary outcomes: length of hospital stay, hospital readmission, 30-day and 90-day all-cause mortality. The present analysis also included surgical site/wound infection as an exploratory infection subtype.
Data items	10b	List and define all other variables for which data were sought (e.g. participant and intervention characteristics, funding sources). Describe any assumptions made about any missing or unclear information.	Methods: Data extraction and risk of bias; Results: Study characteristics; Table 1. Variables included study design, country, cancer site, sample size, iron formulation/dosing, comparator, timing of administration, outcomes and adverse events. Assumptions for imputed infection counts are described in Methods: Outcomes.
Study risk of bias assessment	11	Specify the methods used to assess risk of bias in the included studies, including details of the tool(s) used, how many reviewers assessed each study and whether they worked independently, and if applicable, details of automation tools used in the process.	Methods: Data extraction and risk of bias; Results: Risk of bias; Figures 2 and 3. RoB 2 was used for randomised trials and ROBINS-I for non-randomised comparative studies; assessment was performed independently by two reviewers.

Effect measures	12	Specify for each outcome the effect measure(s) (e.g. risk ratio, mean difference) used in the synthesis or presentation of results.	Methods: Statistical analysis. Risk ratios were used for dichotomous outcomes and mean differences for continuous outcomes.
Synthesis methods	13a	Describe the processes used to decide which studies were eligible for each synthesis (e.g. tabulating the study intervention characteristics and comparing against the planned groups for each synthesis (item #5)).	Methods: Eligibility criteria; Outcomes; Statistical analysis. Outcome-specific inclusion in each main synthesis required at least three studies with extractable arm-level data. For subgroup analyses, subgroup-specific estimates were pooled descriptively when at least two studies contributed data; formal subgroup-difference tests were performed only when both subgroups had at least two studies.
Synthesis methods	13b	Describe any methods required to prepare the data for presentation or synthesis, such as handling of missing summary statistics, or data conversions.	Methods: Outcomes; Statistical analysis. Infection counts were imputed from infective subtypes where necessary; zero cells were handled with a 0.5 continuity correction; median/IQR data were converted using Wan et al. (2014).
Synthesis methods	13c	Describe any methods used to tabulate or visually display results of individual studies and syntheses.	Methods: Data Synthesis and Statistical Analysis; Results: Tables 1–9 and Figures 1–13. Results were displayed using a PRISMA flow diagram, risk-of-bias plots, forest plots, subgroup/sensitivity tables, funnel plots and a GRADE summary table.
Synthesis methods	13d	Describe any methods used to synthesize results and provide a rationale for the choice(s). If meta-analysis was performed, describe the model(s), method(s) to identify the presence and extent of statistical heterogeneity, and software package(s) used.	Methods: Data Synthesis and Statistical Analysis. Random-effects inverse-variance meta-analysis using the DerSimonian–Laird estimator was used; heterogeneity was assessed using τ^2, Cochran's Q and I^2. Analyses were implemented in Python 3 using NumPy and SciPy, with forest plots generated in R using the meta package and funnel plots produced in Python using matplotlib.
Synthesis methods	13e	Describe any methods used to explore possible causes of heterogeneity among study results (e.g. subgroup analysis, meta-regression).	Methods: Sensitivity and Subgroup Analysis; Results: Table 6, Table 7 and Table 8. Heterogeneity was explored using subgroup analyses by cancer site, iron formulation and timing of parenteral iron administration.
Synthesis methods	13f	Describe any sensitivity analyses conducted to assess robustness of the synthesized results.	Methods: Sensitivity and Subgroup Analysis; Results: Sensitivity and subgroup analyses for infection; Tables 2–5 and Table 8; Figures 5 and 6. Analyses included exclusion of imputed events, randomised-trial-only analysis, non-randomised-only analysis, leave-one-out analysis, risk-of-bias sensitivity analysis, length-of-stay direct mean/standard deviation sensitivity analysis, REML with Hartung–Knapp sensitivity analysis, and exploratory timing subgroup analyses.
Reporting bias assessment	14	Describe any methods used to assess risk of bias due to missing results in a synthesis (arising from reporting biases).	Methods: Publication Bias; Results: Reporting of Publication Bias; Figures 12 and 13. Funnel plots and Egger's tests were used for outcomes with sufficient contributing studies; infection assessment was exploratory because k = 9.
Certainty assessment	15	Describe any methods used to assess certainty (or confidence) in the body of evidence for an outcome.	Results: GRADE Summary of Findings; Table 9. GRADE was used to assess certainty/confidence across risk of bias, inconsistency, indirectness, imprecision and publication bias.
**RESULTS**			
Study selection	16a	Describe the results of the search and selection process, from the number of records identified in the search to the number of studies included in the review, ideally using a flow diagram.	Results: Study selection; Figure 1. Reports identified, duplicates removed, records screened, full texts assessed and 14 unique comparative studies included.
Study selection	16b	Cite studies that might appear to meet the inclusion criteria, but which were excluded, and explain why they were excluded.	Figure 1: PRISMA flow diagram gives full-text exclusion categories/reasons. Individual excluded studies are not separately tabulated in the manuscript.
Study characteristics	17	Cite each included study and present its characteristics.	Results: Study characteristics; Table 1. Each included study is cited and summarised by design, cancer site, timing, iron formulation and comparator.
Risk of bias in studies	18	Present assessments of risk of bias for each included study.	Results: Risk of bias; Figures 2 and 3. Risk-of-bias assessments are presented for randomised and non-randomised studies.
Results of individual studies	19	For all outcomes, present, for each study: (a) summary statistics for each group (where appropriate) and (b) an effect estimate and its precision (e.g. confidence/credible interval), ideally using structured tables or plots.	Results: Primary and secondary outcome sections; forest plots Figures 4–11; Tables 2–8. Forest plots present individual study effects and 95% CIs where included in syntheses; subgroup tables list contributing studies.

Results of syntheses	20a	For each synthesis, briefly summarise the characteristics and risk of bias among contributing studies.	Results: Risk of bias; Primary outcome; Secondary outcomes; Tables 1–9. Contributing study characteristics and risk-of-bias context are summarised.
Results of syntheses	20b	Present results of all statistical syntheses conducted. If meta-analysis was done, present for each the summary estimate and its precision (e.g. confidence/credible interval) and measures of statistical heterogeneity. If comparing groups, describe the direction of the effect.	Results: Primary outcome, Sensitivity analyses, Secondary outcomes, Synthesis of findings, Reporting of Publication Bias and GRADE Summary of Findings; Tables 2–9; Figures 4–13. Summary estimates, 95% CIs, P values and heterogeneity are reported.
Results of syntheses	20c	Present results of all investigations of possible causes of heterogeneity among study results.	Results: Table 6 (cancer-site subgroup), Table 7 (iron-formulation subgroup), and Table 8 (timing subgroup).
Results of syntheses	20d	Present results of all sensitivity analyses conducted to assess the robustness of the synthesized results.	Results: Sensitivity and subgroup analyses for infection; Leave-one-out analysis; Length-of-stay sensitivity analysis; Tables 2–5 and Table 8; Figures 5 and 6.
Reporting biases	21	Present assessments of risk of bias due to missing results (arising from reporting biases) for each synthesis assessed.	Results: Reporting of Publication Bias; Figures 12 and 13. Funnel plots and Egger's test results are reported for infection and length of stay.
Certainty of evidence	22	Present assessments of certainty (or confidence) in the body of evidence for each outcome assessed.	Results: GRADE Summary of Findings; Table 9. Certainty of evidence is reported for each assessed outcome.
**DISCUSSION**			
Discussion	23a	Provide a general interpretation of the results in the context of other evidence.	Discussion: first paragraphs interpreting infection, surgical site/wound infection, LOS, readmission, mortality and timing subgroup findings in context of existing evidence.
Discussion	23b	Discuss any limitations of the evidence included in the review.	Limitations: paragraphs addressing observational evidence, risk of bias, varied infection definitions, imputed outcomes, clinical heterogeneity, limited event numbers, and limited reporting of nutritional status, surgical access, ICG use and operative-field contamination.
Discussion	23c	Discuss any limitations of the review processes used.	Limitations: paragraphs addressing imputation, conversion of median/IQR data, inconsistent reporting, limited available outcomes, and inability to meta-analyse variables not consistently reported.
Discussion	23d	Discuss implications of the results for practice, policy, and future research.	Clinical importance; Discussion; Conclusion. Implications for perioperative patient blood management, nutritional optimisation, surgical-pathway factors and future standardised infection reporting are discussed.
**OTHER INFORMATION**			
Registration and protocol	24a	Provide registration information for the review, including register name and registration number, or state that the review was not registered.	Methods: Design and reporting. PROSPERO registration CRD420261289224.
Registration and protocol	24b	Indicate where the review protocol can be accessed, or state that a protocol was not prepared.	Methods: Design and reporting. Protocol/registration details are available in the PROSPERO record CRD420261289224.
Registration and protocol	24c	Describe and explain any amendments to information provided at registration or in the protocol.	No protocol amendments are described in the manuscript. Additional timing and surgical site/wound infection analyses are labelled as exploratory in the revised manuscript.
Support	25	Describe sources of financial or non-financial support for the review, and the role of the funders or sponsors in the review.	Title page/front matter: Funding statement.
Competing interests	26	Declare any competing interests of review authors.	Title page/front matter: Conflict of interest statement.
Availability of data, code and other materials	27	Report which of the following are publicly available and where they can be found: template data collection forms; data extracted from included studies; data used for all analyses; analytic code; any other materials used in the review.	Data Availability Statement / Supplementary Materials. If not already included in the manuscript or journal submission system, add a statement that extracted data and analysis materials are available from the corresponding author upon reasonable request or are provided as supplementary files.

From: Page MJ, McKenzie JE, Bossuyt PM, Boutron I, Hoffmann TC, Mulrow CD, et al. The PRISMA 2020 statement: an updated guideline for reporting systematic reviews. BMJ 2021;372:n71. doi: 10.1136/bmj.n71. This work is licensed under CC BY 4.0. To view a copy of this license, visit https://creativecommons.org/licenses/by/4.0/
